# Response to therapeutic sleep deprivation: a naturalistic study of clinical and genetic factors and post-treatment depressive symptom trajectory

**DOI:** 10.1038/s41386-018-0092-y

**Published:** 2018-05-17

**Authors:** Nina Trautmann, Jerome C. Foo, Josef Frank, Stephanie H. Witt, Fabian Streit, Jens Treutlein, Steffen Conrad von Heydendorff, Maria Gilles, Andreas J. Forstner, Ulrich Ebner-Priemer, Markus M. Nöthen, Michael Deuschle, Marcella Rietschel, Major Depressive Disorder Working Group of the Psychiatric Genomics Consortium

**Affiliations:** 1 Central Institute of Mental Health, Department of Genetic Epidemiology in Psychiatry, Medical Faculty Mannheim, University of Heidelberg, J5 68159 Mannheim, Germany; 2 Central Institute of Mental Health, Department of Psychiatry and Psychotherapy, Medical Faculty Mannheim, University of Heidelberg, J5 68159 Mannheim, Germany; 3 Institute of Human Genetics, University of Bonn School of Medicine & University Hospital Bonn, Sigmund-Freud-Straße 25, 53127 Bonn, Germany; 4 Department of Genomics, Life & Brain Research Center, University of Bonn, Sigmund-Freud-Straße 25, 53127 Bonn, Germany; 5 Department of Psychiatry (UPK), University of Basel, Wilhelm Klein-Strasse 27, 4012 Basel, Switzerland; 6 Human Genomics Research Group, Department of Biomedicine, University of Basel, Hebelstrasse 20, 4031 Basel, Switzerland; 7 Institute of Medical Genetics and Pathology, University Hospital Basel, Schönbeinstrasse 40, 4031 Basel, Switzerland; 8 Department of Sport and Sport Science, Karlsruhe Institute of Technology. Engler-Bunte-Ring 16, 76131 Karlsruhe, Germany

## Abstract

Research has shown that therapeutic sleep deprivation (SD) has rapid antidepressant effects in the majority of depressed patients. Investigation of factors preceding and accompanying these effects may facilitate the identification of the underlying biological mechanisms. This exploratory study aimed to examine clinical and genetic factors predicting response to SD and determine the impact of SD on illness course. Mood during SD was also assessed via visual analogue scale. Depressed inpatients (*n* = 78) and healthy controls (*n* = 15) underwent ~36 h of SD. Response to SD was defined as a score of ≤ 2 on the Clinical Global Impression Scale for Global Improvement. Depressive symptom trajectories were evaluated for up to a month using self/expert ratings. Impact of genetic burden was calculated using polygenic risk scores for major depressive disorder. In total, 72% of patients responded to SD. Responders and non-responders did not differ in baseline self/expert depression symptom ratings, but mood differed. Response was associated with lower age (*p* *=* 0.007) and later age at life-time disease onset (*p* *=* 0.003). Higher genetic burden of depression was observed in non-responders than healthy controls. Up to a month post SD, depressive symptoms decreased in both patients groups, but more in responders, in whom effects were sustained. The present findings suggest that re-examining SD with a greater focus on biological mechanisms will lead to better understanding of mechanisms of depression.

## Introduction

Therapeutic sleep deprivation (SD) reliably induces rapid and substantial antidepressant effects in the majority of patients with a major depressive episode [1–4]. A recent meta-analysis of SD studies showed an average response rate of ~ 50% with significant variability, with up to 78% of patients responding to SD treatment [5]. Although its therapeutic value is limited due to relapse after recovery sleep [2, 6], it has been shown that chronotherapeutic techniques (i.e., sleep phase advance, bright light therapy) affecting circadian machinery can prolong SD effects [7].

SD is particularly unique in its defined immediate positive effect on depressive mood and may therefore offer unique insights about the biological factors underlying depression. Response to SD has been associated with various factors, including circadian rhythms [8–11]; tiredness [12]; disease diagnosis and “endogenous depression” [13–16]; age-related features [17–20], and candidate gene variants [4, 21, 22]. Several plausible hypotheses have been formulated [7, 23–25], but a comprehensive understanding of underlying factors, especially with respect to the biological mechanisms involved, has not yet been achieved.

MDD is a heterogeneous disorder, and it is thought that a multitude of genetic variants are involved in course, development, and response to treatment [26, 27]. Understanding the role of genetic risk in modulation of response to treatment might allow the identification of potential responders, eventually leading to improvements in personalized care. It has been observed that higher genetic burden for psychiatric disorders is associated with response to treatment [28–30].

Recent genome-wide association studies with large samples have made substantial progress with identification of common risk variants for MDD [31, 32]. Furthermore, polygenic risk scores (PRS), which summarize the effects of many single-nucleotide polymorphisms in a single risk score offer the ability to associate burden of disease with clinical and phenotypic factors, and have been successfully applied to explore the genetic architecture of complex disorders [29, 31–34].

In this naturalistic exploratory study, we assessed clinical and genetic factors associated with response to SD, going beyond the study of individual candidate genes for the first time, using all-genomic information in the form of PRS. We also evaluated mood longitudinally during SD, and the impact of SD on the further trajectory of depressive symptoms.

## Materials and methods

### Participants

Seventy-eight inpatients (34 females; age mean ± standard deviation = 43.54 ± 14.80 years) presenting with an episode of major depression (unipolar, *n* = 71; bipolar I, *n* = 6; and bipolar II, *n* = 1) participated in this study. Depression was diagnosed according to ICD-10 criteria. Patients were recruited between August 2013 and April 2015 from consecutive admissions to the depression unit of the Central Institute of Mental Health (CIMH) in Mannheim, Germany. The study protocol stipulated that for 5 + days prior to SD, no changes were allowed to the medication regimen. Prescribed medication included typical and atypical antidepressants, lithium, and adjunct therapies (for details, see supplementary text). Fifteen healthy controls (eight females; 40.53 ± 15.90 years) with no history of psychiatric/somatic disorders were recruited through an online advertisement on the CIMH website. The investigation was carried out in accordance with the latest version of the Declaration of Helsinki and approved by the local ethics committee. All participants provided written informed consent following a detailed explanation of the study.

### SD

**Fig. 1:**
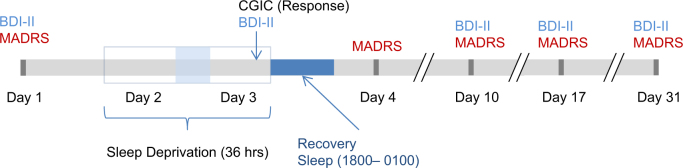
Schematic timeline of study schedule. CGI = Clinical Global Impression; BDI-II = Beck Depression Inventory-II; MADRS = Montgomery-Åsberg Depression Rating Scale

Participation began on Day 1 (see Fig. 1) whereupon baseline variables (see below) were assessed. During Day 2, patients engaged in normal ward routines. SD was conducted in small groups of 1–5 participants under staff supervision. Participants remained awake from ~ 0600 h on Day 2 to 1800 h on Day 3 (36 h). On Day 3, patients engaged in normal ward routines until undergoing recovery sleep from 1800–0100 h. Sleep phase advance was then carried out, shifting sleep 1h  forward each day until the patient’s regular sleep pattern was reached. Controls underwent SD alongside patients; their participation ended after the first recovery sleep.

### Data collection

#### Blood sampling

On Day 1, a venous blood sample was collected from participants for genome-wide genotyping, which was performed using the Global Screening Array (Illumina, Inc., San Diego, CA, USA). Genotyping and quality control procedures are described in detail in the supplement and elsewhere [29, 33].

#### Demographic and clinical characteristics

Day 1 assessments included: demographics, including sex, age, age at initial disease onset (AaO); clinical parameters (body mass index, pulse); history of psychiatric and somatic disorders and family history (FH) of MDD or bipolar disorder (BD).

The validated German version [35] of the Morningness-Eveningness-Questionnaire (D-MEQ) [36] was used to assess circadian rhythm/diurnal variation. The D-MEQ comprises 19 items on circadian patterns, identifying morning, intermediate, and evening chronotypes.

#### Response to SD

Response to SD was evaluated between 1600 h and 1700 h on Day 3 by the senior clinical researcher (MD) using the Clinical Global Impression Scale for Global Improvement (CGIC) [37]. Possible CGIC scores were: 1 = Very much improved; 2 = Much improved; 3 = Minimally improved; 4 = No change; 5 = Minimally worse; 6 = Much worse; 7 = Very much worse. Response and non-response were defined as scores of ≤ 2 and ≥ 3, respectively. The CGIC was chosen as the primary response outcome owing to its utility in measuring immediate response (see supplementary text for details regarding scale choice).

#### Depressive Symptoms Scales

The 10-item Montgomery-Åsberg Depression Rating Scale (MADRS) [38] was completed by the senior clinical researcher (MD) on Days 1, 4, 10, 17, and 30. The 21-item Beck Depression Inventory-II (BDI-II) [39] was completed by patients on Days 1, 3, 10, 17, and 30.

#### During SD

Participants completed visual analogue scales (VAS) [40] for mood every 2 h from 1000 h on Day 2–1800 h on Day 3. Ratings ranged from: “worst mood imaginable (0)” to “best mood imaginable (10)”. Tiredness ratings were also assessed by VAS (see supplementary text). Locomotor activity was acquired using the SOMNOwatch (SOMNOmedics GmbH, Germany), and patients recorded in a wear log when the device was worn/removed; these were inspected to identify subjects who had fallen asleep before response assessment.

### Data analysis

Statistical analyses were performed using IBM SPSS Statistics for Windows version 24. Statistical significance was set at *p* < 0.05.

#### Descriptive statistics

Descriptive statistics were calculated. For continuous variables, mean values were compared using independent samples *t* tests. For nominal values, proportions were compared using Fisher’s exact test.

#### Genotyping and PRS calculation

PRS [34] were calculated using genome-wide association data from the Psychiatric Genomics Consortium MDDII (Cases: *n* = 59,851, Controls: *n* = 113,154) [32]. A *p* value threshold of 1.0 was found to give best-fit (for details, see supplementary text). Scores were standardized to the mean and standard deviation of controls [41]. Binomial logistic regression was used to compare PRS across disease state. To compare PRS across groups (non-responder/responder/control) one-way analysis of variance (ANOVA) was used.

#### Baseline predictors of response to SD

To identify baseline predictors of response to SD, a binomial logistic regression analysis was performed. Response was specified as the dependent variable. Categorical independent variables comprised: sex; diurnal variation (morning/intermediate/evening chronotype); season (spring/summer/autumn/winter); diagnosis (Unipolar MDD/BD); and FH. Continuous independent variables comprised: MDD-PRS; age; AaO; and baseline BDI-II and MADRS scores.

#### Mood and tiredness trajectories

To compare mood trajectories between responders and non-responders during SD, a random-intercepts mixed model was used (accounting for intra-individual clustering of observations). Mood was specified as the dependent variable. MDD-PRS, response, timepoint and the interaction between response × timepoint were specified as fixed factors. Timepoint was centred to midnight and included in a repeated term with an AR1 covariance structure. The same model with tiredness as the dependent variable was specified.

We also tested whether baseline (one-way ANOVA) and mood trajectories (random-intercepts mixed model, fixed effects: diagnosis, timepoint, diagnosis × timepoint interaction) differed between bipolar and unipolar patients.

#### Depressive symptoms score trajectories

Correlations between MADRS and BDI-II scores were examined over all measurement days. Score trajectories were examined using random-intercepts mixed models. Fixed effects included sex, season, diagnosis, response, and measurement day entered as factors. Age, AaO, and MDD-PRS were entered as covariates. The response × measurement day interaction was entered as a fixed effect. Measurement day was included in a repeated term with a diagonal covariance structure.

## Results

### Demographics and descriptive statistics

Descriptive statistics are shown in Table S1. Six patients were excluded from the analysis as they did not complete SD. Four patients were excluded for having fallen asleep prior to response rating. Thus, data from a total of 68 patients were included in the subsequent analyses (except for PRS analysis). A total of 49 (49/68; 72.1%) responded to SD. In total, 5/7 of the bipolar patients responded to SD.

### PRS

**Fig. 2:**
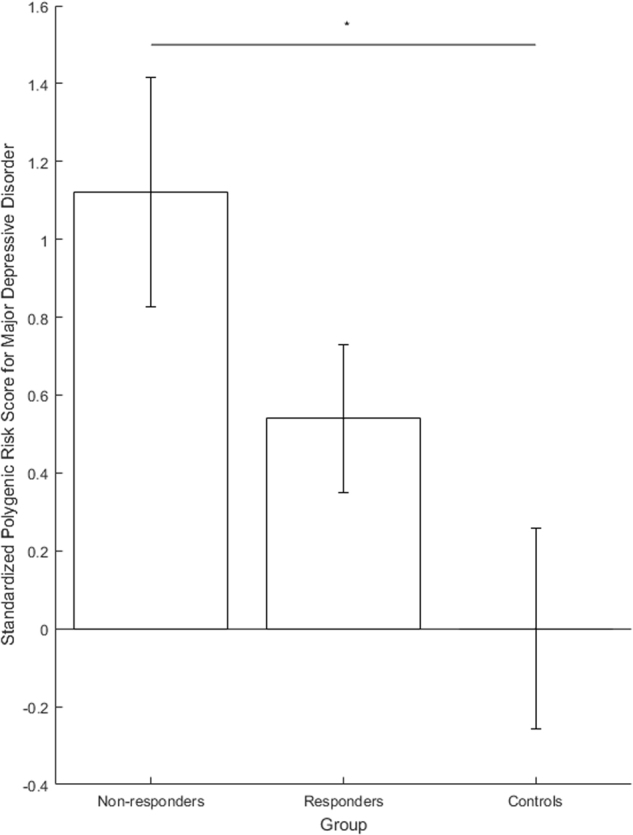
Polygenic Risk Scores (PRS) for major depression in non-responders, responders and healthy controls. Scores are standardized to mean and standard deviation of healthy controls. Error bars denote standard error of mean. ^*^ *p* < 0.05

The regression model comparing PRS for disease state (controls *n* = 15; patients *n* = 72) found higher PRS in patients at the trend level (*p* *=* 0.068, Δ_Nagelkerke_*R*^2^ = 0.066). The ANOVA to compare groups (responders *n* = 46, non-responders n = 18, controls *n* = 15) found a significant difference between groups (*F*_2,76_ = 3.426, *p* = 0.038). A post hoc Tukey test found the group difference to be driven by higher scores in non-responders than controls (significant, *p* = 0.029). Although not significant, higher scores were found in non-responders than responders (*p* = 0.212) and controls than responders (*p* = 0.309) (see Fig. 2 and supplementary text for additional details).

### Baseline predictors of response to SD

The regression model included 57 patients due to missing (assessment or genetic) data (Table S2). The model was statistically significant, *χ*^2^(13) = 24.477, *p* = 0.027, explaining 50.2% of the variance in response. Lower age (*p* = .007) and higher AaO (*p* = 0.003) were significantly associated with an increased likelihood of response. No significant effects were found for PRS (*p* = 0.907); FH (*p* = 0.125); sex (*p* = 0.148); season (*p* = 0.587); baseline BDI-II score (*p* = 0.986); baseline MADRS score (*p* *=* 0.314); diagnosis (*p* = 0.691); or diurnal variation (*p* = 0.343).

### Mood and tiredness

**Fig. 3:**
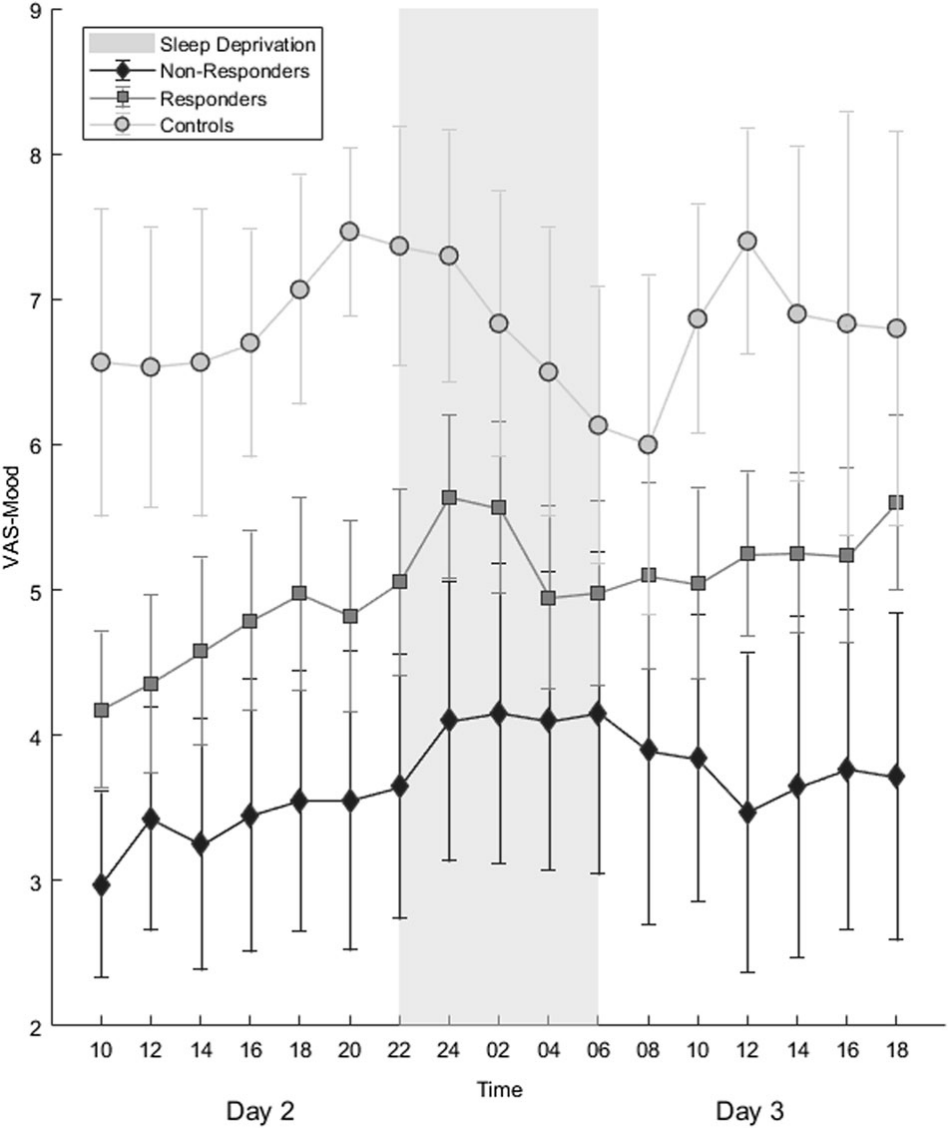
Trajectories of mean mood during sleep deprivation. Error bars denote 95% confidence intervals.

Figure 3 shows trajectories for group mean mood throughout SD.

In the mixed model analysis of mood (Table S3a), significant main effects of timepoint (*F*_16,540.801_ = 2.518, *p* = 0.001) and response (*F*_1,63.217_ = 8.811, *p* = 0.004) were observed. In the whole cohort, mood improved over time (see Table S3a), whereas worse mood was observed in non-responders vs. responders (*t* = –2.109, *df* = 215.848, *p* = 0.036). No significant effects of response × timepoint interaction were observed (*p* = 0.781; only at the final observation point did the interaction show a trend towards significance, *p* = 0.098). No significant effect of MDD-PRS was observed (*p* = 0.276). Estimated correlation between any two consecutive assessment points was significant (AR1 rho, *p* < 0.001).

No significant difference was found in baseline mood between bipolar and unipolar patients (uneven sample sizes, Levene’s statistic: *F*_1,62_ = 3.42, *p* = 0.069; Welch’s Statistic: *F*_1,6.421_ = 0.366, *p* = 0.566). The mixed model found a significant main effect of timepoint (*F*_16,539.414_ = 1.900, *p* = 0.018) but not diagnosis (*F*_1,63.578_ = 0.23, *p* = 0.880) or diagnosis × timepoint interaction (*F*_16,539.414_ = 0.831, *p* = 0.651).

The analysis of tiredness (Table S3b) found only a significant effect of timepoint; (*F*_16,544.059_ = 11.662, *p* < 0.001) participants became increasingly tired as time progressed (see supplementary text for details).

### Depressive symptoms (MADRS and BDI-II)

**Fig. 4:**
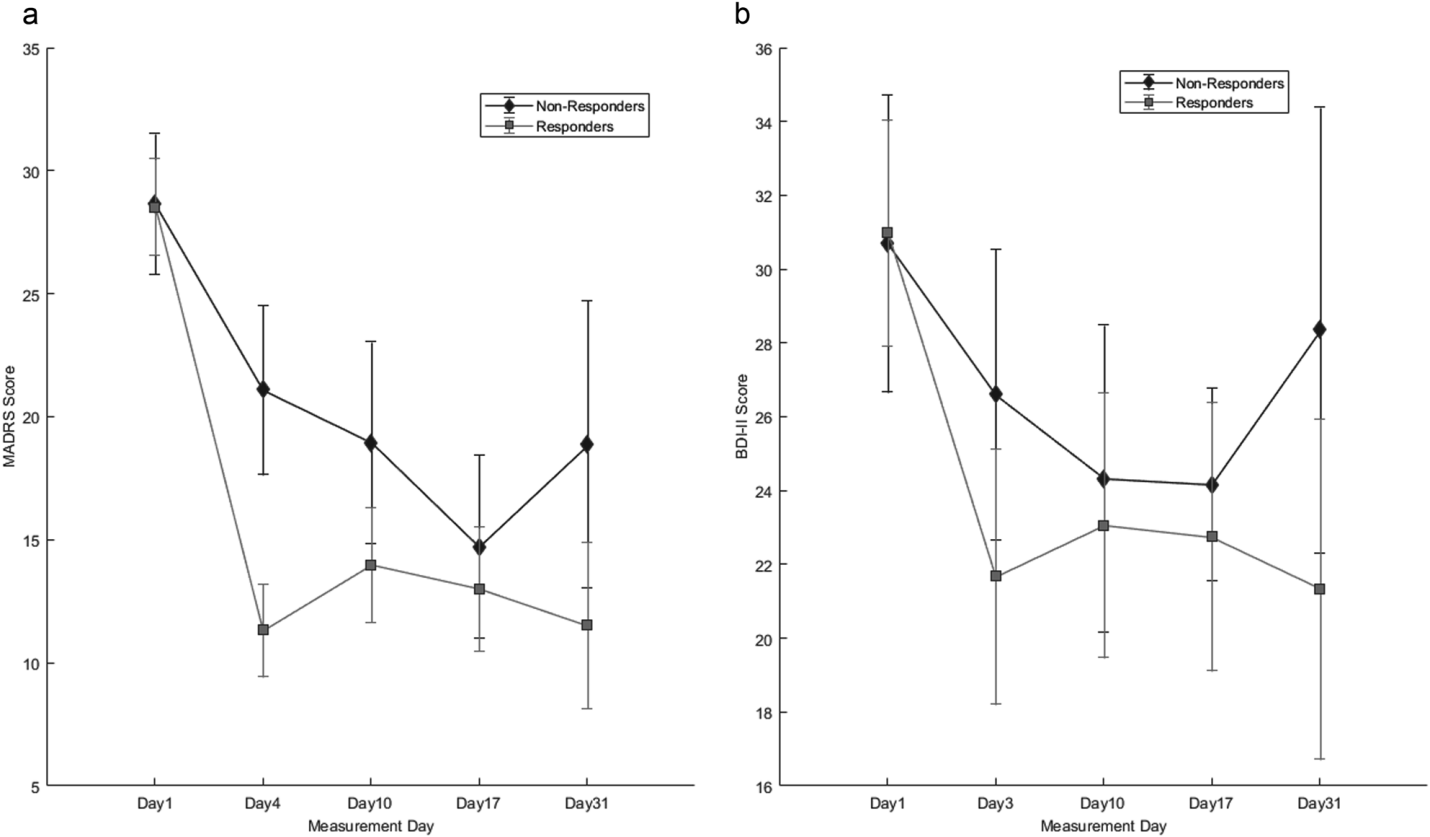
Post-treatment trajectories of **a** MADRS and **b** BDI-II scores. Error bars denote 95% confidence intervals. BDI-II = Beck Depression Inventory-II; MADRS = Montgomery-Åsberg Depression Rating Scale

Responders and non-responders did not differ in terms of baseline MADRS and BDI-II scores (Fig. 4a, b). The correlation between MADRS and BDI-II scores on all measurement days was consistent (all Pearson *r* ≥ 0.4) and significant (all *p* < 0.001) (Table S4a).

For the MADRS, significant main effects were observed for *response* (*F*_1,68,573_ = 6.155, *p* = 0.016); measurement day (*F*_4,87,373_ = 49.388, *p* < 0.001); measurement day × response interaction (*F*_4,87.492_ = 5.339, *p* = 0.001); and season (*F*_3,61.090_ = 3.854, *p* *=* 0.014). MADRS scores significantly decreased on all measurement days compared with baseline (all *p* < 0.001) (Fig. 4a, Table S4b). The interaction term revealed significantly lower scores in responders than non-responders on Days 4 (*t* = 4.242, *df* = 83.491, *p* < 0.001); 10 (*t* = 2.394, *df* = 80.704, *p* = 0.019); and 31(*t* *=* 2.767, *df* = 55.519, *p* = 0.008), but not Day 17 (*t* = 1.169, *df* = 81.646, *p* = 0.246). MADRS scores were significantly higher in spring than during other seasons (vs. summer *p* = 0.013; autumn *p* *=* 0.020; winter *p* = 0.002). No significant effects of sex (*p* = 0.420), age (*p* = 0.519)*,* AaO (*p* = 0.855)*,* FH (*p* = 0.784), or diagnosis (*p* = 0.850) or MDD-PRS (*p* *=* 0.155) were observed.

For BDI-II, significant main effects were observed for measurement day (*F*_4,65.719_ = 13.140, *p* < 0.001), season (*F*_3,57.224_ =  9.733, *p* < 0.001) and sex (*F*_1,56.431_ = 5.091, *p* = 0.028). BDI-II scores decreased significantly on all measurement days compared with baseline (all *p* < 0.001) (Fig. 4b, Table S4c) and significantly higher in spring compared with all other seasons (all *p* < 0.001). No significant interaction between response × measurement day was observed (*F*_4,65.719_ = 65.719, *p* = 0.296, trend for higher scores in non-responders on Day 31, *p* = 0.085). Higher BDI-II scores were observed in women (*t* = 2.256, *df* = 56.431, *p* = 0.28). No significant effects of response (*p* = 0.918), age (*p* = 0.960), AaO (*p* = 0.941), FH (*p* = 0.566), or diagnosis (*p* = 0.712) or MDD-PRS (*p* = 0.559) were observed.

## Discussion

The observed association between response and both younger age at presentation [17, 19] and higher age at disease onset [20] replicate previous reports. The finding that responders and non-responders did not differ in terms of baseline depressive symptom scores is consistent with reports of depression severity not influencing SD response [11, 17, 19, 42]. Previously reported associations with diurnal variation were not observed [8–10].

In the present cohort, the proportion of response to SD was on the higher end of the range reported in a recent meta-analysis, in which response rates ranged from 7 to 78% [5]. The authors hypothesized that the small individual sample sizes were likely to contribute to this wide range of response rates. It is of note that the mean sample size of these studies was ~ 23 and ~ 66% of these studies had smaller sample sizes. In the present study, we applied the same protocol consistently in a large sample of patients over a protracted period of time, making the response rate we observed more robust and less prone to spurious factors which might be observed in small samples assessed during relatively short time spans.

We examined genetic burden for MDD using PRS, finding significantly higher scores in non-responders than controls. We also found higher PRS in non-responders compared to responders, although differences were not statistically significant. These preliminary data suggest that underlying biological differences may be involved in SD effects and may suggest an avenue for exploration in larger samples. Although initial depression severity did not differ in responders and non-responders, differing subjective mood and mood trajectories were observed. Better baseline mood in responders may indicate better attitude towards the treatment, and should be further explored. Interestingly, both responders and non-responders experienced some degree of mood improvement during SD; although the interaction between response and timepoint was not statistically significant (Fig. 3a, S1), this might be qualitatively accounted for by mood scores in responders in crossing the mid-point of the VAS (i.e., from the ‘negative’ to ‘positive’ side of the scale). Further research should use multi-dimensional mood assessments to better examine the changes.

We found no evidence of differing baseline mood/mood trajectories between unipolar and bipolar patients. Nevertheless, care should be taken when assessing mood in bipolar patients, as definitions of “better” mood may differ from unipolar patients if referencing a previous manic/hypomanic episode, leading to potential bias.

Tiredness levels, previously reported to predict response [12], did not differ between responders and non-responders; except for in the early evening (see supplementary text, Figure S2) trajectories were similar in all participants.

Correlations observed between BDI-II/MADRS suggest validity of both scales. Although trajectories appeared similar, the interaction between response and assessment day was significant for MADRS, but not BDI-II. This may be attributable to (1) differences in number of items and points assigned to each item and (2) the fact that the BDI-II is a subjective measure, containing many items assessing maladaptive personality traits [43] unlikely to change in the short-term. Interestingly, women reported higher BDI-II but not MADRS scores than men, which may further suggest that the symptoms contributing to depression are different between the sexes.

Importantly, these longitudinal scores reflect clinical treatment outcomes, suggesting that response to SD may be a general indicator of response to further treatment. We included season to control for possible effects (daylight hours, temperature), finding more pronounced depressive symptoms in the spring, which is consistent with previous research showing exacerbation of mood disorders in spring [44]. We note that whereas the BDI-II and MADRS detected no baseline differences between groups, the VAS did. The VAS measures positive mood, which is not assessed in depressive symptom scales. This suggests that future studies should quantify positive mood, and as mentioned above, that measurement of the multiple dimensions of mood/affect would allow more rigorous characterization of behavioural patterns during SD.

This study had several limitations. First, as this was a naturalistic study, patients were not randomised/stratified with respect to medication, diagnoses, age at onset, or illness duration. Second, the sample size was too small to control for all potential influences, despite being one of the larger reported SD cohorts to date. Third, response to SD was assessed using the CGIC, which does not allow specification of which symptoms have changed. However, changes in both the MADRS and BDI-II scores were consistent with the CGIC. Fourth, for the tiredness measure, participants were not given further instruction beyond that given in the questionnaire to differentiate ‘sleepiness’ from the ‘general fatigue’ characterizing depression, and caution is needed when interpreting this finding. Fifth, comparison with depressed patients not undergoing SD would have strengthened the interpretation of our findings. Finally, we did not correct *p* values for multiple testing.

In conclusion, the rapid, pronounced effects of SD render it a well-controlled, efficient model [45]. We propose that it is a promising context to apply targeted investigation of abnormal clock gene expression related to MDD and SD in humans [46] and animal models [47], novel methods such as genome-wide analyses (of the epi/genome and proteome) [22, 48–53], and furthermore ecologically valid techniques such as ambulatory assessment [54]. We believe that such an approach is suitable to not only link observed phenotypic changes with underlying biological factors, but to do so in a way such that depression heterogeneity (and interindividual differences) can be dissected.

## Electronic supplementary material

Supplementary Material
