# Supplementary material for: Response to therapeutic sleep deprivation: a naturalistic study of clinical and genetic factors and post-treatment depressive symptom trajectory
Source: Neuropsychopharmacology. 2018 May 17;43(13):2572–7. doi: 10.1038/s41386-018-0092-y (PMC6224527; doi:10.1038/s41386-018-0092-y)
Supplement: Supplementary file 1 — Supplementary Material [file 41386_2018_92_MOESM1_ESM.doc]

**Supplementary Information for:**

**Response to Therapeutic Sleep Deprivation: A Naturalistic Study of Clinical and Genetic Factors and Post-Treatment Depressive Symptom Trajectory**

Nina Trautmann^a,b†^, Jerome C. Foo^a†^**^*^**, Josef Frank^a^, Stephanie H. Witt^a^, Fabian Streit^a^, Jens Treutlein^a^, Steffen Conrad von Heydendorff^b^, Maria Gilles^b^, Major Depressive Disorder Working Group of the Psychiatric Genomics Consortium^#^, Andreas J. Forstner^c,d,e,f,g^, Ulrich Ebner-Priemer^h^, Markus M. Nöthen^c,d^, Michael Deuschle^b‡^, Marcella Rietschel^a‡^

^†^ Co-First Authors

^‡^ Co-Last Authors

**^*^**Corresponding author

^#^Members are listed in the supplementary material

**Contents:**

Supplementary Text

Supplementary Tables: **Tables S1 – S5**

Supplementary Figures: **Figures S1 – S3**

Psychiatric Genomics Consortium MDD Working Group Author List and Affiliations

References

**Supplementary Text**

**Genotyping, quality control, and polygenic risk score calculation**

Genome-wide genotyping was performed using the Global Screening Array (Illumina, Inc., San Diego, CA, USA). Genotyping and quality control procedures have been described in detail elsewhere^1,2^. In brief, stringent quality control filtering criteria were applied in order to account for call rates, heterozygosity, population stratification, relatedness, Hardy-Weinberg equilibrium, minor allele frequency (>0.1), and others. The MHC complex of chromosome 6 was excluded. Following the application of these filters, genome wide data were available for n = 212 412 markers.

Polygenic Risk Scores (PRS)^3^ for depression were calculated using PRSice 1.25^4^. Marker weights were calculated using the natural logarithm of odds ratios provided in the results file from Psychiatric Genomics Consortium^5^, which contained 59 851 cases and 113 154 controls. The present sleep deprivation cohort is independent from the PGC-MDD2 sample. Only markers which could be matched to the PGC-MDD2 results file by rs number were used. Clumping was carried out to retain only one representative variant per region of linkage disequilibrium (LD) using thresholds of p1 1, p2 1, an LD threshold of r^2^≥0.1, and a distance threshold of 500kb. Average scores were calculated for a range of *p*-value thresholds (5x10^-8^, 1x10^-6^, 1x10^-4^, 0.001, 0.01, 0.05, 0.1, 0.2, 0.5, 1.0). Best fit (discrimination between patients and controls) was found for a threshold of 1.0, which yielded n=53788 markers (see **Figure S3 and Table S5** for fitting at all thresholds). Scores from this threshold were used in the analyses. PRS were normalised to values between 0 and 1.

A binomial logistic regression analysis was carried out to determine the contribution of PRS to disease state, and also to compare PRS between patients and controls. Disease state (patient/controls) was specified as the dependent variable. In the first block, population stratification variables (first two PCA components and sex) were specified as predictors. In the second block, PRS were entered. The full model gave a Nagelkerke R^2^ of 0.140, while the model without PRS gave a Nagelkerke R^2^ of 0.074, suggesting that PRS contributed of 6.6% to model variability. One-way analysis of variance (ANOVA) was performed to compare controls, responders, and non-responders. The test of homogeneity of variances revealed no significant differences (Levene’s *F*_2,76_ = 0.782, *p* = 0.461). The ANOVA revealed a main effect for group (F_2,76_= 3.426, *p* = 0.038). Post-hoc Tukey tests revealed significantly lower PRS in controls compared to non-responders (*p* = 0.029), but no significant difference between controls and responders (*p* = 0.309). While responders had lower scores than non-responders, this difference was not significant (*p* = 0.212) (see **Figure 1**).

**Medication**

Forty-three patients were prescribed antidepressant therapy only: 1 antidepressant (n = 28); 2 antidepressants (n = 14); or 3 antidepressants (n = 1). Thirteen patients were prescribed an antidepressant plus adjunct therapy in the form of: Quetiapine (n = 10); Aripiprazole (n = 2); Risperidone (n = 1); or Prothipendyl (n = 1). Three patients were prescribed Quetiapine only. Three patients were prescribed Quetiapine plus Aripiprazole. Eight patients were prescribed Lithium and: an antidepressant (n = 3); Quetiapine (n = 1); an antidepressant plus Quetiapine (n = 2); Olanzapine (n = 1); or Amisulpride (n = 1). Six patients were medication-free at the time of SD, and medication was not assessed for one patient who dropped out of the study at an early stage.

**CGI-C as Response Outcome**

Most sleep SD studies have used percent reduction of depression scale scores as response outcome measures. However, these scales contain items assessing ‘daily-life’ symptoms which may not be suitable for assessment of rapid changes, especially in an SD context. For example, scores on the “reduced sleep” MADRS item would be inflated as a result of SD, and items such as “concentration difficulties” or “lassitude” assessing regular activities might reflect sleepiness. As removing these items would affect validity of the composite scale, we decided to use the CGIC following sleep deprivation prior to recovery sleep. The CGIC asks the clinician to “Rate total improvement whether or not, in your judgement, it is due entirely to treatment. Compared to his/her condition at admission to the project, how much has he/she changed?” As it is a relative assessment, it requires comprehensive knowledge about the participant. Thus, in the present study, the senior clinical researcher handled all of the expert assessments for all subjects from the time of entry into the project.

**Additional Information on Statistical Methods**

*Binary Logistic Regression Analysis:*

The Box-Tidwell procedure was used to test the assumption that predictors were linearly related to the log odds. No significant interactions were found between any predictor/natural log. Due to missing assessment and/or genetic data, the regression model included 57 patients only. The model was statistically significant (χ^2^(13) = 24.477, p = 0.027). The model explained 50.2% of the variance in response and correctly classified 78.9% of cases (sensitivity=90.2%; specificity=50.0%).

*Mood and Tiredness*

Subjects completed VAS assessments every two hours from 1000hrs on Day2 to 1800hrs on Day 3 (**Figures S1, S2**). This resulted in 17 assessment points. Analysis of mood included data from 1027 observations while analysis of tiredness included data from 1026 observations.

On the VAS, *Tiredness* ratings ranged from: “not tired at all” to “so tired that it's hard to stay awake”. The subjects were not given any instructions to differentiate ‘sleepiness’ from the ‘general fatigue’ associated with depression, so interpretation of these results require caution.

A random-intercepts mixed model was used with *Tiredness* specified as the dependent variable. *PRS for MDD*, *Response* and *Timepoint* and the interaction between *Response*Timepoint* were specified as fixed factors. *Timepoint* was centred to midnight and included in a repeated term with an AR1 covariance structure. The analysis **(Table S3b)** found only a significant effect of *timepoint* (*F*_16,544.059_ = 11.662, *p* < 0.001); participants became increasingly tired as time progressed. No significant effect of *response* (*p* = 0.542) or overall *response*timepoint* interaction (*p* = 0.355) was observed, but examining the interaction term revealed lower tiredness scores in non-responders than responders at times 1600 (*p* = 0.040), 1800 (*p* = 0.061) and 2000 (*p* = 0.048). No significant association was observed with PRS (*p* = 0.389).

*MADRS and BDI-II*

Analysis for MADRS included scores from 275 observations while analysis for BDI included scores from 269 observations. While the number of non-responders remained approximately equal throughout the 30-day study period (i.e. these individuals remained inpatients), a number of responders were discharged due to an improvement in their mental state (**Table S1**, duration of hospital stay post SD, and **Table S4a**). This suggests the need for caution when interpreting results, but also supports the idea that response is associated with better outcomes.

**Table S1. Descriptive and Clinical Statistics for Responders, Non-responders, and Control Groups**

|  |  | **Responders**  **(n = 49)** | **Non-responders**  **(n = 19)** | **Significance** ^2^ | **Controls**  **(n = 15)** |
| --- | --- | --- | --- | --- | --- |
| *Characteristic* | | *Mean (SD)* | *Mean (SD)* |  | *Mean (SD)* |
| Age (years) | | 43.08 (14.53) | 42.84 (13.92) | 0.951 | 40.53 (15.90) |
| Age at Disorder Onset (years) | | 35.08 (13.63) | 25.74 (10.08) | **0.002^**^** | / |
| Body Mass Index | | 26.63 (6.39) | 26.92 (4.50) | 0.855 | 25.31 (4.6) |
| Pulse (beats per minute) | | 77.02 (13.40) | 79.95 (13.18) | 0.42 | 75.43 (9.88) |
| Baseline MADRS | | 28.53 (6.98) | 28.47 (6.64) | 0.976 | 2.13 (1.96) |
| Baseline BDI-II | | 30.6 (10.95) | 31.53 (8.79) | 0.719 | 4.13 (3.56) |
| D-MEQ | | 48.54 (12.17) | 48 (7.64) | 0.834 | 53.67 (7.67) |
| Total duration of hospital stay | | 50.49 (34.92) | 64.32 (42.58) | 0.173 | / |
| Duration of hospital stay post sleep deprivation | | 35.67 (24.35) | 49.84 (36.13) | **0.066 ^Δ^** | / |
|  |  |  |  |  |  |
|  |  | **n (%)** | **n (%)** | **Significance ^3^** | **n (%)** |
| Male / Female | | 31/18 (63.27/36.73) | 9/10 (47.37/52.63) | 0.278 | 7/8 (46.67/53.33) |
| MDD / BD | | 44/5 (89.8 / 10.2) | 17/2 (89.47/10.53) | 1 | / |
| FH | | 24/25 (48.98) | 9/10 (47.37) | 1 | 1 (6.67) |
| Morning type ^1^ | | 9 (19.57) | 2 (11.76) |  | 3 (20) |
| Evening type ^1^ | | 13 (28.26) | 6 (35.29) |  | / |
| Intermediate type ^1^ | | 24 (52.17) | 9 (52.94) |  | 12 (80) |
| BD: Bipolar Disorder; BDI-II: Beck Depression Inventory II; D-MEQ: German version of the Morningness-Eveningness-Questionnaire; FH: Family History of Psychiatric Disorder; MADRS: Montgomery-Åsberg Depression Rating Scale; SD = Standard deviation, MDD: Major Depressive Disorder.  ^1^ Calculated from D-MEQ scores  ^2^ t-test for independent samples, two-tailed  ^3^ Fisher's exact test, two-tailed  ^Δ^ *p* < 0.1, ^**^ *p* < 0.01 | | | | | |

**Table S2. Baseline Predictors of Response to Sleep De**privation Treatment

|  | **B** | **Std. Error** | **Wald** | ***p*** | **OR** | **95% CI for OR** | |
| --- | --- | --- | --- | --- | --- | --- | --- |
|  |  |  |  |  |  | Lower | Upper |
| Male Sex | 1.506 | 1.042 | 2.091 | 0.148 | 4.510 | 0.586 | 34.745 |
| Age | -0.183 | 0.068 | 7.265 | **0.007**^**^ | 0.833 | 0.729 | 0.951 |
| Age at Onset | 0.270 | 0.092 | 8.610 | **0.003**^**^ | 1.309 | 1.094 | 1.568 |
| FH | 1.518 | 0.989 | 2.358 | 0.125 | 4.565 | 0.657 | 31.701 |
| Bipolar Disorder | 0.670 | 1.684 | 0.159 | 0.691 | 1.955 | 0.072 | 52.991 |
| Intermediate Type ^1^ |  |  | 2.138 | 0.343 |  |  |  |
| Morning Type | 1.796 | 1.237 | 2.106 | 0.147 | 6.023 | 0.533 | 68.065 |
| Evening Type | 0.633 | 1.107 | 0.328 | 0.567 | 1.884 | 0.215 | 16.488 |
| Spring ^a^ |  |  | 1.940 | 0.585 |  |  |  |
| Summer | -0.164 | 1.923 | 0.007 | 0.932 | 0.848 | 0.020 | 36.751 |
| Autumn | -1.722 | 1.599 | 1.160 | 0.282 | 0.179 | 0.008 | 4.106 |
| Winter | -0.783 | 1.545 | 0.256 | 0.613 | 0.457 | 0.022 | 9.452 |
| MADRS1 | 0.079 | 0.078 | 1.015 | 0.314 | 1.082 | 0.928 | 1.262 |
| BDI1 | -0.001 | 0.051 | 0.000 | 0.986 | 0.999 | 0.904 | 1.104 |
| Polygenic Risk Score | 0.329 | 2.823 | 0.014 | 0.907 | 1.389 | 0.005 | 351.262 |
| Constant | -2.786 | 3.894 | 0.512 | 0.474 | 0.062 |  |  |

B*:* Unstandardized coefficient; BDI-II1: Baseline Beck Depression Inventory-II; CI: Confidence Interval; FH: Family History of Major Depressive Disorder or Bipolar Disorder; MADRS1: Baseline Montgomery-Åsberg Depression Rating Scale; OR: Odds Ratio; SE: Standard Error.

^1^ Reference

^**^ p < 0.01

**Table S3a. Mixed Model Analysis of Mood: Type III Tests of Fixed Effects**

| **Source** | **Numerator df** | **Denominator df** | **F** | ***p*** |
| --- | --- | --- | --- | --- |
| Intercept | 1 | 62.071 | 53.826 | **0.000^***^** |
| Response | 1 | 63.217 | 8.811 | **0.004^**^** |
| Timepoint | 16 | 540.801 | 2.518 | **0.001^**^** |
| Polygenic Risk Score for MDD | 1 | 61.846 | 1.207 | 0.276 |
| Response * Timepoint | 16 | 540.803 | 0.714 | 0.781 |

df: degrees of freedom; MDD: Major Depressive Disorder

^***^ p < 0.001, ^**^ p < 0.01

**Table S3b. Mixed Model Analysis of Mood: Estimates of Fixed Effects**

| **Parameter** | **Estimate** | **Std. Error** | **df** | **t** | ***p*** |
| --- | --- | --- | --- | --- | --- |
| Intercept | 3.711463 | 0.537580 | 89.794 | 6.904 | **0.000**^***^ |
| Response ^1^ |  |  |  |  |  |
| No Response | -1.243606 | 0.589798 | 215.848 | -2.109 | **0.036**^*^ |
| Day 2 1000 ^1^ |  |  |  |  |  |
| Day 2 1200 | 0.084856 | 0.350745 | 396.261 | 0.242 | 0.809 |
| Day 2 1400 | 0.340312 | 0.350690 | 397.197 | 0.970 | 0.332 |
| Day 2 1600 | 0.630372 | 0.350576 | 398.764 | 1.798 | **0.073^Δ^** |
| Day 2 1800 | 0.724391 | 0.351420 | 405.475 | 2.061 | **0.040**^*^ |
| Day 2 2000 | 0.522562 | 0.349720 | 404.437 | 1.494 | 0.136 |
| Day 2 2200 | 0.774771 | 0.348143 | 404.134 | 2.225 | **0.027**^*^ |
| Day 2 2400 | 1.365680 | 0.347907 | 409.904 | 3.925 | **0.000**^***^ |
| Day 3 0200 | 1.263408 | 0.347487 | 419.234 | 3.636 | **0.000**^***^ |
| Day 3 0400 | 0.683862 | 0.346736 | 434.292 | 1.972 | **0.049**^*^ |
| Day 3 0600 | 0.740680 | 0.345396 | 458.614 | 2.144 | **0.033**^*^ |
| Day 3 0800 | 0.814080 | 0.344453 | 502.222 | 2.363 | **0.018**^*^ |
| Day 3 1000 | 0.774508 | 0.340673 | 564.688 | 2.273 | **0.023**^*^ |
| Day 3 1200 | 1.050590 | 0.333063 | 665.316 | 3.154 | **0.002**^**^ |
| Day 3 1400 | 1.128903 | 0.320322 | 817.859 | 3.524 | **0.000**^***^ |
| Day 3 1600 | 1.066251 | 0.293422 | 955.928 | 3.634 | **0.000**^***^ |
| Day 3 1800 | 1.437525 | 0.238140 | 875.255 | 6.036 | **0.000**^***^ |
| Polygenic Risk Score for MDD | 1.160486 | 1.056114 | 61.846 | 1.099 | 0.276 |
| Day 2 1000 * Response ^1^ |  |  |  |  |  |
| Day 2 1200 * Response | 0.331811 | 0.646018 | 393.285 | 0.514 | 0.608 |
| Day 2 1400 * Response | 0.048577 | 0.645971 | 393.903 | 0.075 | 0.940 |
| Day 2 1600 * Response | -0.019261 | 0.645878 | 394.929 | -0.030 | 0.976 |
| Day 2 1800 * Response | -0.113280 | 0.646282 | 397.826 | -0.175 | 0.861 |
| Day 2 2000 * Response | 0.227438 | 0.645261 | 399.053 | 0.352 | 0.725 |
| Day 2 2200 * Response | 0.058562 | 0.644233 | 401.473 | 0.091 | 0.928 |
| Day 2 2400 * Response | -0.143458 | 0.643794 | 407.229 | -0.223 | 0.824 |
| Day 3 0200 * Response | 0.069926 | 0.643011 | 416.539 | 0.109 | 0.913 |
| Day 3 0400 * Response | 0.593916 | 0.641615 | 431.568 | 0.926 | 0.355 |
| Day 3 0600 * Response | 0.703764 | 0.639119 | 455.850 | 1.101 | 0.271 |
| Day 3 0800 * Response | 0.352586 | 0.635439 | 496.460 | 0.555 | 0.579 |
| Day 3 1000 * Response | 0.076185 | 0.632561 | 567.674 | 0.120 | 0.904 |
| Day 3 1200 * Response | -0.670436 | 0.625381 | 674.194 | -1.072 | 0.284 |
| Day 3 1400 * Response | -0.626900 | 0.602129 | 815.660 | -1.041 | 0.298 |
| Day 3 1600 * Response | -0.382340 | 0.552139 | 954.403 | -0.692 | 0.489 |
| Day 3 1800 * Response | -0.749727 | 0.452303 | 876.998 | -1.658 | **0.098^Δ^** |
| All Timepoints * No Response ^1^ |  |  |  |  |  |

df: degrees of freedom; MDD: Major Depressive Disorder

1. Reference
2. ^***^ p < 0.001, ^**^ p < 0.01, ^*^ p < 0.05, **^Δ^** p < 0.10

**Table S3c.** Mixed Model Analysis of Mood: Type III Tests of Fixed Effects

| **Source** | **Numerator df** | **Denominator df** | **F** | ***p*** |
| --- | --- | --- | --- | --- |
| Intercept | 1 | 62.180 | 43.493 | **0.000**^***^ |
| Response | 1 | 63.639 | 0.377 | 0.542 |
| Timepoint | 16 | 544.059 | 11.662 | **0.000**^***^ |
| Polygenic Risk Score for MDD | 1 | 61.910 | 0.753 | 0.389 |
| Response * Timepoint | 16 | 544.063 | 1.096 | 0.355 |

df: degrees of freedom; MDD: Major Depressive Disorder

^***^ p < 0.001

**Table 3d.** Mixed Model Analysis of Tiredness: Estimates of Fixed Effects

| **Parameter** | **Estimate** | **Std. Error** | **df** | **t** | ***p*** |
| --- | --- | --- | --- | --- | --- |
| Intercept | 3.181572 | 0.647936 | 93.912 | 4.910 | **0.000**^***^ |
| Response ^1^ |  |  |  |  |  |
| No Response | 0.099195 | 0.726212 | 235.250 | 0.137 | 0.891 |
| Day 2 1000 ^1^ |  |  |  |  |  |
| Day 2 1200 | -0.069368 | 0.450144 | 362.684 | -0.154 | 0.878 |
| Day 2 1400 | 0.429214 | 0.450061 | 363.930 | 0.954 | 0.341 |
| Day 2 1600 | 0.205888 | 0.449898 | 365.950 | 0.458 | 0.647 |
| Day 2 1800 | 0.195748 | 0.450843 | 372.784 | 0.434 | 0.664 |
| Day 2 2000 | 0.357810 | 0.448780 | 373.259 | 0.797 | 0.426 |
| Day 2 2200 | 0.276518 | 0.446915 | 375.533 | 0.619 | 0.536 |
| Day 2 2400 | 0.696973 | 0.446437 | 383.255 | 1.561 | 0.119 |
| Day 3 0200 | 0.731064 | 0.445628 | 395.197 | 1.641 | 0.102 |
| Day 3 0400 | 1.924246 | 0.444259 | 413.687 | 4.331 | **0.000**^***^ |
| Day 3 0600 | 2.390155 | 0.441938 | 442.445 | 5.408 | **0.000**^***^ |
| Day 3 0800 | 2.006103 | 0.439761 | 491.554 | 4.562 | **0.000**^***^ |
| Day 3 1000 | 1.529849 | 0.433735 | 561.171 | 3.527 | **0.000**^***^ |
| Day 3 1200 | 1.961529 | 0.422426 | 668.995 | 4.643 | **0.000**^***^ |
| Day 3 1400 | 2.120136 | 0.404131 | 824.963 | 5.246 | **0.000**^***^ |
| Day 3 1600 | 1.938096 | 0.367895 | 957.448 | 5.268 | **0.000**^***^ |
| Day 3 1800 | 2.302861 | 0.296867 | 882.330 | 7.757 | **0.000**^***^ |
| Polygenic Risk Score for MDD | 1.091113 | 1.257501 | 61.910 | 0.868 | 0.389 |
| Day 2 1000 * Response ^1^ |  |  |  |  |  |
| Day 2 1200 * Response | -0.375077 | 0.827344 | 357.921 | -0.453 | 0.651 |
| Day 2 1400 * Response | -0.595880 | 0.827253 | 358.874 | -0.720 | 0.472 |
| Day 2 1600 * Response | -1.705888 | 0.827086 | 360.387 | -2.063 | **0.040**^*^ |
| Day 2 1800 * Response | -1.556859 | 0.827470 | 363.828 | -1.881 | **0.061^Δ^** |
| Day 2 2000 * Response | -1.635587 | 0.826126 | 366.211 | -1.980 | **0.048**^*^ |
| Day 2 2200 * Response | -1.109852 | 0.824739 | 370.371 | -1.346 | 0.179 |
| Day 2 2400 * Response | -0.585862 | 0.823846 | 378.043 | -0.711 | 0.477 |
| Day 3 0200 * Response | 0.185603 | 0.822336 | 389.912 | 0.226 | 0.822 |
| Day 3 0400 * Response | 0.270199 | 0.819781 | 408.298 | 0.330 | 0.742 |
| Day 3 0600 * Response | -0.390155 | 0.815449 | 436.918 | -0.478 | 0.633 |
| Day 3 0800 * Response | -0.228326 | 0.809042 | 483.007 | -0.282 | 0.778 |
| Day 3 1000 * Response | -0.490978 | 0.802755 | 560.963 | -0.612 | 0.541 |
| Day 3 1200 * Response | -0.167554 | 0.790268 | 675.394 | -0.212 | 0.832 |
| Day 3 1400 * Response | 0.048447 | 0.756924 | 821.893 | 0.064 | 0.949 |
| Day 3 1600 * Response | 0.505740 | 0.689305 | 955.964 | 0.734 | 0.463 |
| Day 3 1800 * Response | 0.871693 | 0.559742 | 880.248 | 1.557 | 0.120 |
| All Timepoints * No Response ^1^ |  |  |  |  |  |

df: degrees of freedom; MDD: Major Depressive Disorder

^1^ Reference

^***^ p < 0.001, ^**^ p < 0.01, ^*^ p < 0.05, **^Δ^** p < 0.10

**Table S4a. Patients completing MADRS/BDI-II Assessments and Correlation between Assessment S**cores

| **Assessment Day** | **MADRS (n)** | | **BDI-II (n)** | | **Correlation of Scores** |
| --- | --- | --- | --- | --- | --- |
|  | **No Response** | **Response** | **No Response** | **Response** | **(Pearson r)** |
| Baseline | 19 | 49 | 19 | 47 | **0.400^***^** |
| Day 3 ^1^ / 4 ^2^ | 19 | 49 | 19 | 46 | **0.537^***^** |
| Day 10 | 18 | 48 | 18 | 47 | **0.474^***^** |
| Day 17 | 17 | 43 | 17 | 40 | **0.546^***^** |
| Day 31 | 15 | 28 | 16 | 27 | **0.566^***^** |
| BDI-II: Beck Depression Inventory-II; MADRS: Montgomery-Åsberg Depression Rating Scale  ^1^ BDI-II, ^2^ MADRS, ^***^ *p* < 0.001 | | | | | |

**Table S4b. Mixed Model for Montgomery-Åsberg Depression Rating Scale Score Trajectories: Type III Tests of Fixed Effects**

| **Source** | **Numerator df** | **Denominator df** | **F** | ***p*** |
| --- | --- | --- | --- | --- |
| Intercept | 1 | 61.523 | 37.402 | **0.000**^***^ |
| Sex | 1 | 60.726 | 0.660 | 0.420 |
| Diagnosis | 1 | 61.565 | 0.036 | 0.850 |
| Measurement Day | 4 | 87.373 | 49.388 | **0.000**^***^ |
| Season | 3 | 61.090 | 3.854 | **0.014**^*^ |
| Response | 1 | 68.573 | 6.155 | **0.016**^*^ |
| Age | 1 | 60.745 | 0.421 | 0.519 |
| Age at Disease Onset | 1 | 60.811 | 0.034 | 0.855 |
| Polygenic Risk Score for MDD | 1 | 61.975 | 2.076 | 0.155 |
| Measurement Day * Response | 4 | 87.492 | 5.339 | **0.001**^**^ |
| FH | 1 | 60.733 | 0.076 | 0.784 |

df: Degrees of Freedom; FH: Family History of Major Depressive Disorder or Bipolar Disorder; MDD: Major Depressive Disorder

^***^ *p* < 0.001, ^**^ *p* < 0.01, ^*^ *p* < 0.05

**Table S4c. Mixed Model for MADRS** Score Trajectories: Estimates of Fixed Effects

| **Parameter**   | **Estimate** | **Std. Error** | **df** | **t** | ***p*** |  | | --- | --- | --- | --- | --- | --- | | Intercept | 33.256985 | 3.846591 | 63.909 | 8.646 | **0.000**^***^ | | Male ^1^ |  |  |  |  |  | | Female | 1.160173 | 1.428598 | 60.726 | 0.812 | 0.420 | | BP ^1^ |  |  |  |  |  | | MDD | -0.403345 | 2.126317 | 61.565 | -0.190 | 0.850 | | Day 1^1^ |  |  |  |  |  | | Day 4 | -17.022727 | 1.122738 | 83.491 | -15.162 | **0.000**^***^ | | Day 10 | -14.553003 | 1.286009 | 80.623 | -11.316 | **0.000**^***^ | | Day 17 | -15.932310 | 1.372117 | 81.427 | -11.611 | **0.000**^***^ | | Day 31 | -17.682011 | 2.000164 | 52.069 | -8.840 | **0.000**^***^ | | Spring ^1^ |  |  |  |  |  | | Summer | -5.574555 | 2.182731 | 61.497 | -2.554 | **0.013**^*^ | | Autumn | -4.684012 | 1.955427 | 60.476 | -2.395 | **0.020**^*^ | | Winter | -6.113951 | 1.895256 | 60.893 | -3.226 | **0.002**^**^ | | Response |  |  |  |  |  | | No Response | -1.067027 | 2.180361 | 71.799 | -0.489 | 0.626 | | Age | -0.053964 | 0.083190 | 60.745 | -0.649 | 0.519 | | Age at Disease Onset | -0.018955 | 0.102946 | 60.811 | -0.184 | 0.855 | | Polygenic Risk Score for MDD | 5.549271 | 3.851171 | 61.975 | 1.441 | 0.155 | | No Response * Day 1 ^1^ |  |  |  |  |  | | No Response * Day 4 | 9.022727 | 2.126763 | 83.491 | 4.242 | **0.000**^***^ | | No Response * Day 10 | 5.884402 | 2.458290 | 80.709 | 2.394 | **0.019**^*^ | | No Response * Day 17 | 2.993552 | 2.559915 | 81.646 | 1.169 | 0.246 | | No Response * Day 31 | 9.420885 | 3.404985 | 55.519 | 2.767 | **0.008**^**^ | | Response * All Days ^1^ |  |  |  |  |  | | No FH | 0.383333 | 1.392840 | 60.733 | 0.275 | 0.784 | | FH ^1^ |  |  |  |  |  | |  |  |
| --- | --- | --- | --- | --- | --- | --- | --- | --- | --- | --- | --- | --- | --- | --- | --- | --- | --- | --- | --- | --- | --- | --- | --- | --- | --- | --- | --- | --- | --- | --- | --- | --- | --- | --- | --- | --- | --- | --- | --- | --- | --- | --- | --- | --- | --- | --- | --- | --- | --- | --- | --- | --- | --- | --- | --- | --- | --- | --- | --- | --- | --- | --- | --- | --- | --- | --- | --- | --- | --- | --- | --- | --- | --- | --- | --- | --- | --- | --- | --- | --- | --- | --- | --- | --- | --- | --- | --- | --- | --- | --- | --- | --- | --- | --- | --- | --- | --- | --- | --- | --- | --- | --- | --- | --- | --- | --- | --- | --- | --- | --- | --- | --- | --- | --- | --- | --- | --- | --- | --- | --- | --- | --- | --- | --- | --- | --- | --- | --- | --- | --- | --- | --- | --- | --- | --- | --- | --- | --- | --- | --- | --- | --- | --- | --- | --- | --- | --- | --- | --- | --- | --- | --- | --- | --- | --- | --- | --- | --- | --- | --- | --- | --- | --- | --- | --- | --- | --- | --- | --- | --- |
|
| BP: Bipolar Disorder; df: Degrees of Freedom; FH: Family History of Major Depressive Disorder or Bipolar Disorder; MDD: Major Depressive Disorder  ^1^ Reference  ^***^ *p* < 0.001, ^**^ *p* < 0.01, ^*^ *p* < 0.05 |  |  |
|  |  |  |
|  |  |  |
|  |  |  |
|  |  |  |
|  |  |  |
|  |  |  |
| **Table S4d. Mixed Model for BDI-II Score Trajectories: Type III Tests of Fixed Effects** |  |  |
| | **Source** | **Numerator df** | **Denominator df** | **F** | ***p*** | | --- | --- | --- | --- | --- | | Intercept | 1 | 56.931 | 34.147 | **0.000**^***^ | | Sex | 1 | 56.431 | 5.091 | **0.028**^*^ | | Diagnosis | 1 | 58.703 | 0.137 | 0.712 | | Measurement Day | 4 | 65.719 | 13.140 | **0.000**^***^ | | Season | 3 | 57.224 | 9.733 | **0.000**^***^ | | Response | 1 | 58.170 | 0.663 | 0.419 | | Age | 1 | 56.573 | 0.003 | 0.960 | | Age at Disease Onset | 1 | 57.044 | 0.006 | 0.941 | | Polygenic Risk Score for MDD | 1 | 58.288 | 0.345 | 0.559 | | Measurement Day * Response | 4 | 65.719 | 1.256 | 0.296 | | FH | 1 | 56.772 | 0.333 | 0.566 | |  |  |
| df: Degrees of Freedom; FH: Family History of Major Depressive Disorder or Bipolar Disorder; MDD: Major Depressive Disorder  ^***^ *p* < 0.001, ^**^ *p* < 0.01, ^*^ *p* < 0.05 |  |  |
|  |  |  |
|  |  |  |
|  |  |  |
|  |  |  |
|  |  |  |
|  |  |  |
|  |  |  |
|  |  |  |
|  |  |  |
|  |  |  |
|  |  |  |
| **Table S4e. Mixed Model for BDI-II Score Trajectories: Type III Tests of Fixed Effects**   | **Parameter** | **Estimate** | **Std. Error** | **df** | **t** | **p** | | --- | --- | --- | --- | --- | --- | | Intercept | 41.122917 | 5.530492 | 59.265 | 7.436 | **0.000**^***^ | | Male ^1^ |  |  |  |  |  | | Female | 4.638274 | 2.055761 | 56.431 | 2.256 | **0.028**^*^ | | BP ^1^ |  |  |  |  |  | | MDD | -1.141314 | 3.079001 | 58.703 | -0.371 | 0.712 | | Day 1 ^1^ |  |  |  |  |  | | Day 3 | -9.430407 | 1.476178 | 73.625 | -6.388 | **0.000**^***^ | | Day 10 | -8.305112 | 1.510273 | 76.543 | -5.499 | **0.000**^***^ | | Day 17 | -8.965920 | 1.549086 | 84.337 | -5.788 | **0.000**^***^ | | Day 31 | -11.085212 | 2.061549 | 59.418 | -5.377 | **0.000**^***^ | | Spring ^1^ |  |  |  |  |  | | Summer | -14.272585 | 3.155368 | 58.268 | -4.523 | **0.000**^***^ | | Autumn | -11.982382 | 2.828033 | 56.956 | -4.237 | **0.000**^***^ | | Winter | -12.967633 | 2.739864 | 57.209 | -4.733 | **0.000**^***^ | | Response ^1^ |  |  |  |  |  | | No Response | -0.308051 | 2.981601 | 70.066 | -0.103 | 0.918 | | Age | -0.006040 | 0.119773 | 56.573 | -0.050 | 0.960 | | Age at Disease Onset | -0.011026 | 0.148458 | 57.044 | -0.074 | 0.941 | | Polygenic Risk Score for MDD | 3.261144 | 5.554265 | 58.288 | 0.587 | 0.559 | | No Response * Day 1 ^1^ |  |  |  |  |  | | No Response * Day 4 | 4.018642 | 2.737112 | 72.528 | 1.468 | 0.146 | | No Response * Day 10 | 1.356948 | 2.848897 | 76.483 | 0.476 | 0.635 | | No Response * Day 17 | 0.205255 | 2.836459 | 83.806 | 0.072 | 0.942 | | No Response * Day 31 | 6.061898 | 3.461403 | 62.940 | 1.751 | **0.085 ^Δ^** | | Response * All Days ^1^ |  |  |  |  |  | | FH ^1^ |  |  |  |  |  | | No FH | -1.157258 | 2.004665 | 56.772 | -0.577 | 0.566 | |  |  |
| BP: Bipolar Disorder; df: Degrees of Freedom; FH: Family History of Major Depressive Disorder or Bipolar Disorder; MDD: Major Depressive Disorder  ^1^ Reference  ^***^ *p* < 0.001, ^*^ *p* < 0.05, **^Δ^** p < 0.10 |  |  |
|  |  |  |

**Figure S1 Mood Trajectories of Responders and Non-Responders Centred to Individual Baselines**


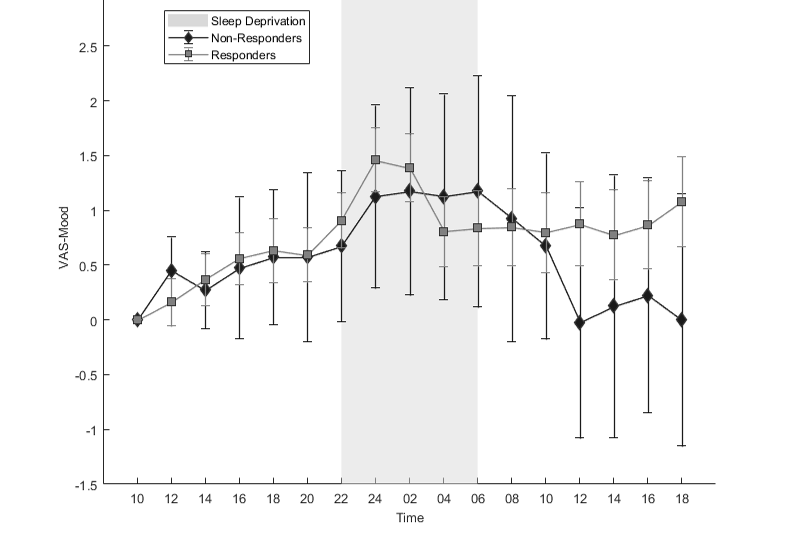


SD: Sleep Deprivation (Error bars denote 95% CI), VAS: Visual Analogue Scale

**Figure S2 Tiredness Trajectories in Responders, Non-Responders and Controls**


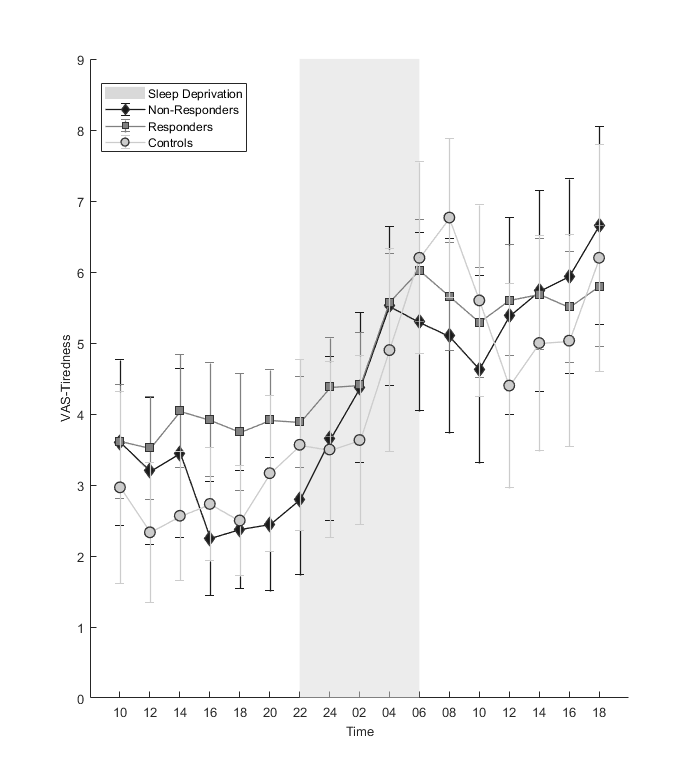


Day 2

Day 3

VAS: Visual Analogue Scale, Error Bars Denote 95% CI

**Figure S3 Model fit for Polygenic Risk Scores at all thresholds**

PRS: Polygenic Risk Scores; #: p < 0.10

**Table S5 Model fit, significance and # of SNPs for PRS at all thresholds**

| P_T_ | Nagelkerke's R^2^ | p-value | # of SNPs |
| --- | --- | --- | --- |
| 0.00000005 | 0 | 0.879 | 7 |
| 0.000001 | 0.017 | 0.324 | 12 |
| 0.0001 | 0 | 0.900 | 97 |
| 0.001 | 0.011 | 0.432 | 463 |
| 0.01 | 0.011 | 0.427 | 2461 |
| 0.05 | 0.007 | 0.538 | 8207 |
| 0.1 | 0.006 | 0.564 | 13466 |
| 0.2 | 0.013 | 0.387 | 21585 |
| 0.5 | 0.053 | 0.100 | 38468 |
| 1 | 0.066 | 0.068^#^ | 53788 |

P_T_: P-value threshold; SNPs: single nucleotide polymorphisms; #: p < 0.10

**Major Depressive Disorder Working Group of the Psychiatric Genomics Consortium**

Naomi R Wray* 1, 2

Stephan Ripke* 3, 4, 5

Manuel Mattheisen* 6, 7, 8, 9

Maciej Trzaskowski* 1

Enda M Byrne 1

Abdel Abdellaoui 10

Mark J Adams 11

Esben Agerbo 9, 12, 13

Tracy M Air 14

Till F M Andlauer 15, 16

Silviu-Alin Bacanu 17

Marie Bækvad-Hansen 9, 18

Aartjan T F Beekman 19

Tim B Bigdeli 17, 20

Elisabeth B Binder 15, 21

Douglas H R Blackwood 11

Julien Bryois 22

Henriette N Buttenschøn 8, 9, 23

Jonas Bybjerg-Grauholm 9, 18

Na Cai 24, 25

Enrique Castelao 26

Jane Hvarregaard Christensen 7, 8, 9

Toni-Kim Clarke 11

Jonathan R I Coleman 27

Lucía Colodro-Conde 28

Baptiste Couvy-Duchesne 2, 29

Nick Craddock 30

Gregory E Crawford 31, 32

Gail Davies 33

Ian J Deary 33

Franziska Degenhardt 34, 35

Eske M Derks 28

Nese Direk 36, 37

Conor V Dolan 10

Erin C Dunn 38, 39, 40

Thalia C Eley 27

Valentina Escott-Price 41

Farnush Farhadi Hassan Kiadeh 42

Hilary K Finucane 43,44

Jerome Foo 45

Andreas J Forstner 34, 35, 46, 47

Josef Frank 45

Héléna A Gaspar 27

Michael Gill 48

Fernando S Goes 49

Scott D Gordon 28

Jakob Grove 7, 8, 9, 50

Lynsey S Hall 11, 51

Christine Søholm Hansen 9, 18

Thomas F Hansen 52, 53, 54

Stefan Herms 34, 35, 47

Ian B Hickie 55

Per Hoffmann 34, 35, 47

Georg Homuth 56

Carsten Horn 57

Jouke-Jan Hottenga 10

David M Hougaard 9, 18

Marcus Ising 58

Rick Jansen 19, 19

Ian Jones 59

Lisa A Jones 60

Eric Jorgenson 61

James A Knowles 62

Isaac S Kohane 63, 64, 65

Julia Kraft 4

Warren W. Kretzschmar 66

Jesper Krogh 67

Zoltán Kutalik 68, 69

Yihan Li 66

Penelope A Lind 28

Donald J MacIntyre 70, 71

Dean F MacKinnon 49

Robert M Maier 2

Wolfgang Maier 72

Jonathan Marchini 73

Hamdi Mbarek 10

Patrick McGrath 74

Peter McGuffin 27

Sarah E Medland 28

Divya Mehta 2, 75

Christel M Middeldorp 10, 76, 77

Evelin Mihailov 78

Yuri Milaneschi 19, 19

Lili Milani 78

Francis M Mondimore 49

Grant W Montgomery 1

Sara Mostafavi 79, 80

Niamh Mullins 27

Matthias Nauck 81, 82

Bernard Ng 80

Michel G Nivard 10

Dale R Nyholt 83

Paul F O'Reilly 27

Hogni Oskarsson 84

Michael J Owen 59

Jodie N Painter 28

Carsten Bøcker Pedersen 9, 12, 13

Marianne Giørtz Pedersen 9, 12, 13

Roseann E. Peterson 17, 85

Erik Pettersson 22

Wouter J Peyrot 19

Giorgio Pistis 26

Danielle Posthuma 86, 87

Jorge A Quiroz 88

Per Qvist 7, 8, 9

John P Rice 89

Brien P. Riley 17

Margarita Rivera 27, 90

Saira Saeed Mirza 36

Robert Schoevers 91

Eva C Schulte 92, 93

Ling Shen 61

Jianxin Shi 94

Stanley I Shyn 95

Engilbert Sigurdsson 96

Grant C B Sinnamon 97

Johannes H Smit 19

Daniel J Smith 98

Hreinn Stefansson 99

Stacy Steinberg 99

Fabian Streit 45

Jana Strohmaier 45

Katherine E Tansey 100

Henning Teismann 101

Alexander Teumer 102

Wesley Thompson 9, 53, 103, 104

Pippa A Thomson 105

Thorgeir E Thorgeirsson 99

Matthew Traylor 106

Jens Treutlein 45

Vassily Trubetskoy 4

André G Uitterlinden 107

Daniel Umbricht 108

Sandra Van der Auwera 109

Albert M van Hemert 110

Alexander Viktorin 22

Peter M Visscher 1, 2

Yunpeng Wang 9, 53, 104

Bradley T. Webb 111

Shantel Marie Weinsheimer 9, 53

Jürgen Wellmann 101

Gonneke Willemsen 10

Stephanie H Witt 45

Yang Wu 1

Hualin S Xi 112

Jian Yang 2, 113

Futao Zhang 1

Volker Arolt 114

Bernhard T Baune 14

Klaus Berger 101

Dorret I Boomsma 10

Sven Cichon 34, 47, 115, 116

Udo Dannlowski 114

EJC de Geus 10, 117

J Raymond DePaulo 49

Enrico Domenici 118

Katharina Domschke 119

Tõnu Esko 5, 78

Hans J Grabe 109

Steven P Hamilton 120

Caroline Hayward 121

Andrew C Heath 89

Kenneth S Kendler 17

Stefan Kloiber 58, 122, 123

Glyn Lewis 124

Qingqin S Li 125

Susanne Lucae 58

Pamela AF Madden 89

Patrik K Magnusson 22

Nicholas G Martin 28

Andrew M McIntosh 11, 33

Andres Metspalu 78, 126

Ole Mors 9, 127

Preben Bo Mortensen 8, 9, 12, 13

Bertram Müller-Myhsok 15, 16, 128

Merete Nordentoft 9, 129

Markus M Nöthen 34, 35

Michael C O'Donovan 59

Sara A Paciga 130

Nancy L Pedersen 22

Brenda WJH Penninx 19

Roy H Perlis 38, 131

David J Porteous 105

James B Potash 132

Martin Preisig 26

Marcella Rietschel 45

Catherine Schaefer 61

Thomas G Schulze 45, 93, 133, 134, 135

Jordan W Smoller 38, 39, 40

Kari Stefansson 99, 136

Henning Tiemeier 36, 137, 138

Rudolf Uher 139

Henry Völzke 102

Myrna M Weissman 74, 140

Thomas Werge 9, 53, 141

Cathryn M Lewis* 27, 142

Douglas F Levinson* 143

Gerome Breen* 27, 144

Anders D Børglum* 7, 8, 9

Patrick F Sullivan* 22, 145, 146

1, Institute for Molecular Bioscience, The University of Queensland, Brisbane, QLD, AU

2, Queensland Brain Institute, The University of Queensland, Brisbane, QLD, AU

3, Analytic and Translational Genetics Unit, Massachusetts General Hospital, Boston, MA, US

4, Department of Psychiatry and Psychotherapy, Universitätsmedizin Berlin Campus Charité Mitte, Berlin, DE

5, Medical and Population Genetics, Broad Institute, Cambridge, MA, US

6, Centre for Psychiatry Research, Department of Clinical Neuroscience, Karolinska Institutet, Stockholm, SE

7, Department of Biomedicine, Aarhus University, Aarhus, DK

8, iSEQ, Centre for Integrative Sequencing, Aarhus University, Aarhus, DK

9, iPSYCH, The Lundbeck Foundation Initiative for Integrative Psychiatric Research,, DK

10, Dept of Biological Psychology & EMGO+ Institute for Health and Care Research, Vrije Universiteit Amsterdam, Amsterdam, NL

11, Division of Psychiatry, University of Edinburgh, Edinburgh, GB

12, Centre for Integrated Register-based Research, Aarhus University, Aarhus, DK

13, National Centre for Register-Based Research, Aarhus University, Aarhus, DK

14, Discipline of Psychiatry, University of Adelaide, Adelaide, SA, AU

15, Department of Translational Research in Psychiatry, Max Planck Institute of Psychiatry, Munich, DE

16, Munich Cluster for Systems Neurology (SyNergy), Munich, DE

17, Department of Psychiatry, Virginia Commonwealth University, Richmond, VA, US

18, Center for Neonatal Screening, Department for Congenital Disorders, Statens Serum Institut, Copenhagen, DK

19, Department of Psychiatry, Vrije Universiteit Medical Center and GGZ inGeest, Amsterdam, NL

20, Virginia Institute for Psychiatric and Behavior Genetics, Richmond, VA, US

21, Department of Psychiatry and Behavioral Sciences, Emory University School of Medicine, Atlanta, GA, US

22, Department of Medical Epidemiology and Biostatistics, Karolinska Institutet, Stockholm, SE

23, Department of Clinical Medicine, Translational Neuropsychiatry Unit, Aarhus University, Aarhus, DK

24, Human Genetics, Wellcome Trust Sanger Institute, Cambridge, GB

25, Statistical genomics and systems genetics, European Bioinformatics Institute (EMBL-EBI), Cambridge, GB

26, Department of Psychiatry, University Hospital of Lausanne, Prilly, Vaud, CH

27, Social Genetic and Developmental Psychiatry Centre, King's College London, London, GB

28, Genetics and Computational Biology, QIMR Berghofer Medical Research Institute, Brisbane, QLD, AU

29, Centre for Advanced Imaging, The University of Queensland, Brisbane, QLD, AU

30, Psychological Medicine, Cardiff University, Cardiff, GB

31, Center for Genomic and Computational Biology, Duke University, Durham, NC, US

32, Department of Pediatrics, Division of Medical Genetics, Duke University, Durham, NC, US

33, Centre for Cognitive Ageing and Cognitive Epidemiology, University of Edinburgh, Edinburgh, GB

34, Institute of Human Genetics, University of Bonn, Bonn, DE

35, Life&Brain Center, Department of Genomics, University of Bonn, Bonn, DE

36, Epidemiology, Erasmus MC, Rotterdam, Zuid-Holland, NL

37, Psychiatry, Dokuz Eylul University School Of Medicine, Izmir, TR

38, Department of Psychiatry, Massachusetts General Hospital, Boston, MA, US

39, Psychiatric and Neurodevelopmental Genetics Unit (PNGU), Massachusetts General Hospital, Boston, MA, US

40, Stanley Center for Psychiatric Research, Broad Institute, Cambridge, MA, US

41, Neuroscience and Mental Health, Cardiff University, Cardiff, GB

42, Bioinformatics, University of British Columbia, Vancouver, BC, CA

43, Department of Epidemiology, Harvard T.H. Chan School of Public Health, Boston, MA, US

44, Department of Mathematics, Massachusetts Institute of Technology, Cambridge, MA, US

45, Department of Genetic Epidemiology in Psychiatry, Central Institute of Mental Health,  Medical Faculty Mannheim, Heidelberg University, Mannheim, Baden-Württemberg, DE

46, Department of Psychiatry (UPK), University of Basel, Basel, CH

47, Human Genomics Research Group, Department of Biomedicine, University of Basel, Basel, CH

48, Department of Psychiatry, Trinity College Dublin, Dublin, IE

49, Psychiatry & Behavioral Sciences, Johns Hopkins University, Baltimore, MD, US

50, Bioinformatics Research Centre, Aarhus University, Aarhus, DK

51, Institute of Genetic Medicine, Newcastle University, Newcastle upon Tyne, GB

52, Danish Headache Centre, Department of Neurology, Rigshospitalet, Glostrup, DK

53, Institute of Biological Psychiatry, Mental Health Center Sct. Hans, Mental Health Services Capital Region of Denmark, Copenhagen, DK

54, iPSYCH, The Lundbeck Foundation Initiative for Psychiatric Research, Copenhagen, DK

55, Brain and Mind Centre, University of Sydney, Sydney, NSW, AU

56, Interfaculty Institute for Genetics and Functional Genomics, Department of Functional Genomics, University Medicine and Ernst Moritz Arndt University Greifswald, Greifswald, Mecklenburg-Vorpommern, DE

57, Roche Pharmaceutical Research and Early Development, Pharmaceutical Sciences, Roche Innovation Center Basel, F. Hoffmann-La Roche Ltd, Basel, CH

58, Max Planck Institute of Psychiatry, Munich, DE

59, MRC Centre for Neuropsychiatric Genetics and Genomics, Cardiff University, Cardiff, GB

60, Department of Psychological Medicine, University of Worcester, Worcester, GB

61, Division of Research, Kaiser Permanente Northern California, Oakland, CA, US

62, Psychiatry & The Behavioral Sciences, University of Southern California, Los Angeles, CA, US

63, Department of Biomedical Informatics, Harvard Medical School, Boston, MA, US

64, Department of Medicine, Brigham and Women's Hospital, Boston, MA, US

65, Informatics Program, Boston Children's Hospital, Boston, MA, US

66, Wellcome Trust Centre for Human Genetics, University of Oxford, Oxford, GB

67, Department of Endocrinology at Herlev University Hospital, University of Copenhagen, Copenhagen, DK

68, Institute of Social and Preventive Medicine (IUMSP), University Hospital of Lausanne, Lausanne, VD, CH

69, Swiss Institute of Bioinformatics, Lausanne, VD, CH

70, Division of Psychiatry, Centre for Clinical Brain Sciences, University of Edinburgh, Edinburgh, GB

71, Mental Health, NHS 24, Glasgow, GB

72, Department of Psychiatry and Psychotherapy, University of Bonn, Bonn, DE

73, Statistics, University of Oxford, Oxford, GB

74, Psychiatry, Columbia University College of Physicians and Surgeons, New York, NY, US

75, School of Psychology and Counseling, Queensland University of Technology, Brisbane, QLD, AU

76, Child and Youth Mental Health Service, Children's Health Queensland Hospital and Health Service, South Brisbane, QLD, AU

77, Child Health Research Centre, University of Queensland, Brisbane, QLD, AU

78, Estonian Genome Center, University of Tartu, Tartu, EE

79, Medical Genetics, University of British Columbia, Vancouver, BC, CA

80, Statistics, University of British Columbia, Vancouver, BC, CA

81, DZHK (German Centre for Cardiovascular Research), Partner Site Greifswald, University Medicine, University Medicine Greifswald, Greifswald, Mecklenburg-Vorpommern, DE

82, Institute of Clinical Chemistry and Laboratory Medicine, University Medicine Greifswald, Greifswald, Mecklenburg-Vorpommern, DE

83, Institute of Health and Biomedical Innovation, Queensland University of Technology, Brisbane, QLD, AU

84, Humus, Reykjavik, IS

85, Virginia Institute for Psychiatric & Behavioral Genetics, Virginia Commonwealth University, Richmond, VA, US

86, Clinical Genetics, Vrije Universiteit Medical Center, Amsterdam, NL

87, Complex Trait Genetics, Vrije Universiteit Amsterdam, Amsterdam, NL

88, Solid Biosciences, Boston, MA, US

89, Department of Psychiatry, Washington University in Saint Louis School of Medicine, Saint Louis, MO, US

90, Department of Biochemistry and Molecular Biology II, Institute of Neurosciences, Center for Biomedical Research, University of Granada, Granada, ES

91, Department of Psychiatry, University of Groningen, University Medical Center Groningen, Groningen, NL

92, Department of Psychiatry and Psychotherapy, Medical Center of the University of Munich, Campus Innenstadt, Munich, DE

93, Institute of Psychiatric Phenomics and Genomics (IPPG), Medical Center of the University of Munich, Campus Innenstadt, Munich, DE

94, Division of Cancer Epidemiology and Genetics, National Cancer Institute, Bethesda, MD, US

95, Behavioral Health Services, Kaiser Permanente Washington, Seattle, WA, US

96, Faculty of Medicine, Department of Psychiatry, University of Iceland, Reykjavik, IS

97, School of Medicine and Dentistry, James Cook University, Townsville, QLD, AU

98, Institute of Health and Wellbeing, University of Glasgow, Glasgow, GB

99, deCODE Genetics / Amgen, Reykjavik, IS

100, College of Biomedical and Life Sciences, Cardiff University, Cardiff, GB

101, Institute of Epidemiology and Social Medicine, University of Münster, Münster, Nordrhein-Westfalen, DE

102, Institute for Community Medicine, University Medicine Greifswald, Greifswald, Mecklenburg-Vorpommern, DE

103, Department of Psychiatry, University of California, San Diego, San Diego, CA, US

104, KG Jebsen Centre for Psychosis Research, Norway Division of Mental Health and Addiction, Oslo University Hospital, Oslo, NO

105, Medical Genetics Section, CGEM, IGMM, University of Edinburgh, Edinburgh, GB

106, Clinical Neurosciences, University of Cambridge, Cambridge, GB

107, Internal Medicine, Erasmus MC, Rotterdam, Zuid-Holland, NL

108, Roche Pharmaceutical Research and Early Development, Neuroscience, Ophthalmology and Rare Diseases Discovery & Translational Medicine Area, Roche Innovation Center Basel, F. Hoffmann-La Roche Ltd, Basel, CH

109, Department of Psychiatry and Psychotherapy, University Medicine Greifswald, Greifswald, Mecklenburg-Vorpommern, DE

110, Department of Psychiatry, Leiden University Medical Center, Leiden, NL

111, Virginia Institute for Psychiatric & Behavioral Genetics, Virginia Commonwealth University, Richmond, VA, US

112, Computational Sciences Center of Emphasis, Pfizer Global Research and Development, Cambridge, MA, US

113, Institute for Molecular Bioscience; Queensland Brain Institute, The University of Queensland, Brisbane, QLD, AU

114, Department of Psychiatry, University of Münster, Münster, Nordrhein-Westfalen, DE

115, Institute of Medical Genetics and Pathology, University Hospital Basel, University of Basel, Basel, CH

116, Institute of Neuroscience and Medicine (INM-1), Research Center Juelich, Juelich, DE

117, Amsterdam Public Health Institute, Vrije Universiteit Medical Center, Amsterdam, NL

118, Centre for Integrative Biology, Università degli Studi di Trento, Trento, Trentino-Alto Adige, IT

119, Department of Psychiatry and Psychotherapy, Medical Center, University of Freiburg, Faculty of Medicine, University of Freiburg, Freiburg, DE

120, Psychiatry, Kaiser Permanente Northern California, San Francisco, CA, US

121, Medical Research Council Human Genetics Unit, Institute of Genetics and Molecular Medicine, University of Edinburgh, Edinburgh, GB

122, Department of Psychiatry, University of Toronto, Toronto, ON, CA

123, Centre for Addiction and Mental Health, Toronto, ON, CA

124, Division of Psychiatry, University College London, London, GB

125, Neuroscience Therapeutic Area, Janssen Research and Development, LLC, Titusville, NJ, US

126, Institute of Molecular and Cell Biology, University of Tartu, Tartu, EE

127, Psychosis Research Unit, Aarhus University Hospital, Risskov, Aarhus, DK

128, University of Liverpool, Liverpool, GB

129, Mental Health Center Copenhagen, Copenhagen Universtity Hospital, Copenhagen, DK

130, Human Genetics and Computational Biomedicine, Pfizer Global Research and Development, Groton, CT, US

131, Psychiatry, Harvard Medical School, Boston, MA, US

132, Psychiatry, University of Iowa, Iowa City, IA, US

133, Department of Psychiatry and Behavioral Sciences, Johns Hopkins University, Baltimore, MD, US

134, Department of Psychiatry and Psychotherapy, University Medical Center Göttingen, Goettingen, Niedersachsen, DE

135, Human Genetics Branch, NIMH Division of Intramural Research Programs, Bethesda, MD, US

136, Faculty of Medicine, University of Iceland, Reykjavik, IS

137, Child and Adolescent Psychiatry, Erasmus MC, Rotterdam, Zuid-Holland, NL

138, Psychiatry, Erasmus MC, Rotterdam, Zuid-Holland, NL

139, Psychiatry, Dalhousie University, Halifax, NS, CA

140, Division of Epidemiology, New York State Psychiatric Institute, New York, NY, US

141, Department of Clinical Medicine, University of Copenhagen, Copenhagen, DK

142, Department of Medical & Molecular Genetics, King's College London, London, GB

143, Psychiatry & Behavioral Sciences, Stanford University, Stanford, CA, US

144, NIHR Maudsley Biomedical Research Centre, King's College London, London, GB

145, Genetics, University of North Carolina at Chapel Hill, Chapel Hill, NC, US

146, Psychiatry, University of North Carolina at Chapel Hill, Chapel Hill, NC, US

**References:**

1. Frank J, Lang M, Witt SH, et al. Identification of increased genetic risk scores for schizophrenia in treatment-resistant patients. *Mol Psychiatry.* 2015;20(7):913.

2. Schizophrenia Working Group of the Psychiatric Genomics C. Biological insights from 108 schizophrenia-associated genetic loci. *Nature.* 2014;511(7510):421-427.

3. Wray NR, Lee SH, Mehta D, Vinkhuyzen AA, Dudbridge F, Middeldorp CM. Research review: Polygenic methods and their application to psychiatric traits. *Journal of Child Psychology and Psychiatry.* 2014;55(10):1068-1087.

4. Euesden J, Lewis CM, O'Reilly PF. PRSice: Polygenic Risk Score software. *Bioinformatics.* 2015;31(9):1466-1468.

5. Wray NR, Ripke S, Mattheisen M, et al. Genome-wide association analyses identify 44 risk variants and refine the genetic architecture of major depression. *Nat Genet.* 2018.
